# Impact of long-term storage and freeze-thawing on eight circulating microRNAs in plasma samples

**DOI:** 10.1371/journal.pone.0227648

**Published:** 2020-01-14

**Authors:** Pamela R. Matias-Garcia, Rory Wilson, Veronika Mussack, Eva Reischl, Melanie Waldenberger, Christian Gieger, Gabriele Anton, Annette Peters, Andrea Kuehn-Steven

**Affiliations:** 1 Research Unit of Molecular Epidemiology, Helmholtz Zentrum Muenchen, German Center for Environmental Health, Neuherberg, Germany; 2 Institute of Epidemiology, Helmholtz Zentrum Muenchen, German Research Center for Environmental Health, Neuherberg, Germany; 3 TUM School of Medicine, Technical University of Munich, Munich, Germany; 4 Department of Animal Physiology and Immunology, TUM School of Life Sciences Weihenstephan, Technical University of Munich (TUM), Freising, Germany; 5 German Center for Cardiovascular Research (DZHK), partner site Munich Heart Alliance, Munich, Germany; 6 German Center for Infection Research (DZIF), partner site Munich, Munich, Germany; Stellenbosch University Faculty of Medicine and Health Sciences, SOUTH AFRICA

## Abstract

Sample collection, processing, storage and isolation methods constitute pre-analytic factors that can influence the quality of samples used in research and clinical practice. With regard to biobanking practices, a critical point in the sample’s life chain is storage, particularly long-term storage. Since most studies examine the influence of different temperatures (4°C, room temperature) or delays in sample processing on sample quality, there is only little information on the effects of long-term storage at ultra-low (vapor phase of liquid nitrogen) temperatures on biomarker levels. Among these biomarkers, circulating miRNAs hold great potential for diagnosis or prognosis for a variety of diseases, like cancer, infections and chronic diseases, and are thus of high interest in several scientific questions. We therefore investigated the influence of long-term storage on levels of eight circulating miRNAs (miR-103a-3p, miR-191-5p, miR-124-3p, miR-30c-5p, miR-451a, miR-23a-3p, miR-93-5p, miR-24-3p, and miR-33b-5p) from 10 participants from the population-based cohort study KORA. Sample collection took place during the baseline survey S4 and the follow-up surveys F4 and FF4, over a time period spanning from 1999 to 2014. The influence of freeze-thaw (f/t) cycles on miRNA stability was also investigated using samples from volunteers (n = 6). Obtained plasma samples were profiled using Exiqon’s miRCURY^TM^ real-time PCR profiling system, and repeated measures ANOVA was used to check for storage or f/t effects. Our results show that detected levels of most of the studied miRNAs showed no statistically significant changes due to storage at ultra-low temperatures for up to 17 years; miR-451a levels were altered due to contamination during sampling. Freeze-thawing of one to four cycles showed an effect only on miR-30c-5p. Our results highlight the robustness of this set of circulating miRNAs for decades of storage at ultra-low temperatures and several freeze-thaw cycles, which makes our findings increasingly relevant for research conducted with biobanked samples.

## Introduction

The pre-analytical phase of studies conducted using biobanking samples consists of the collection, retrieval, processing and transport of biological samples. All steps can heavily influence the integrity of samples and, later, the results of analyses [1]. Since most studies examine the influence of different temperatures (4°C, room temperature) or delays in sample processing on sample quality, there is only little information on the effects of long-term storage at ultra-low (vapor phase of liquid nitrogen) temperatures on biomarker levels.

Circulating microRNAs (miRNAs) are found among these biomarkers. MiRNAs are 22–25 nucleotide-long, small non-coding ribonucleic acids that function as post-transcriptional regulators in gene expression by targeting specific messenger RNA (mRNA) [2]. These miRNA-mRNA interactions can lead to either gene downregulation, by either repressing the translation or completely degrading the targeted mRNA [3]; or to a positive regulation, by enhancing translation in a process termed as RNA activation (RNAa) [4]. As listed in miRBase (release 21) (www.mirbase.org/), the human genome is currently thought to code for 1881 precursors accounting and for 2588 mature miRNAs [5], each participating in the regulation of a great number of target mRNAs and hundreds of gene targets [6], which underlines their potential influence on almost every genetic pathway [7–9]. MiRNA research conducted during the last decade has hinted at their potential to be minimally invasive diagnostic, prognostic and predictive biomarkers [10] in a variety of fields, amongst which cancer [11], clinical trials [12], and infectious diseases [13] are a few examples.

For the aforementioned reasons, circulating miRNAs are of high interest in several scientific questions. In order for the results from any miRNA analysis to be valid, recommendations on how to increase miRNA stability and reproducibility should be followed [14–16]. Moreover, the influence of parameters like study design and data analysis [17], pre-analytic conditions [18–21] and miRNA profiling technique [22–25] should be taken into account, as each individual step in the methodological procedure can potentially have major impact on miRNA detection.

In terms of sample collection and its impact on miRNA stability, miRNAs in serum and plasma have been shown to be stable for up to 48 h even at room temperature or after multiple freeze–thaw cycles [23, 26]. It has also been shown that circulating blood miRNAs can withstand degradation by ribonucleases (RNases) through inclusion in cell-derived extracellular vesicles or by binding to transporter proteins, such as Argonaute 2 and high-density lipoproteins [10, 27, 28].

Although many studies have examined the impact of different temperatures (4°C, room temperature: RT), and differences or delays in sample processing / time-to-freeze on sample quality [1, 21, 23, 29–34], a critical period in the sample’s life chain that has not been sufficiently studied is long-term storage (years or even decades) at ultra-low temperatures (e.g. in vapor phase of liquid nitrogen). Moreover, the impact of several freeze-thaw cycles on sample integrity is also of high interest for biomarker research.

Thus, we investigated the impact of long-term storage on the integrity of eight different miRNAs either used as markers of sample quality of plasma samples or having expression levels in plasma known to be abundant enough for confident detection [35, 36]. Plasma samples, one of the most common sample types in biomarker research due to ease of accessibility, were collected during three surveys of the KORA study (baseline and two follow-ups) from the same participants and stored up to a 17 years. Additionally, we analyzed the influence of four freeze-thaw cycles on a different set of samples. The overall aim of the study was to assess the stability and integrity of a set of eight miRNAs isolated from plasma samples after many years of storage at ultra-low temperatures and, further, the influence of repeated freeze-thawing on the chosen circulating miRNAs. Both questions are highly relevant for evaluating the reliability of biobank plasma samples and the reproducibility of results based on the investigated miRNAs.

## Materials and methods

### Study design

The KORA study (Cooperative health research in the Region of Augsburg) is an independent population-based cohort study from the general population living in the region of Augsburg, Southern Germany. The KORA S4 prospective cohort conducted from 1999 to 2001 including 4,021 participants. A first follow-up (F4) was conducted from 2006 to 2008 with 3,080 participants in total; the KORA FF4 survey was a second follow-up of S4 and was conducted in 2013/2014 with 2,279 participants. More information about the KORA study is available under: https://www.helmholtz-muenchen.de/kora/fuer-wissenschaftler/ueberblick-kora-studien/studienuebersicht/index.html and published by Holle et al. [37].

### Ethical considerations

The study has been conducted according to the principles expressed in the Declaration of Helsinki [38]. Written informed consent has been given by each participant. The study was reviewed and approved by the local ethics committee (Bayerische Landesaerztekammer).

### Sample selection

**Fig 1:**
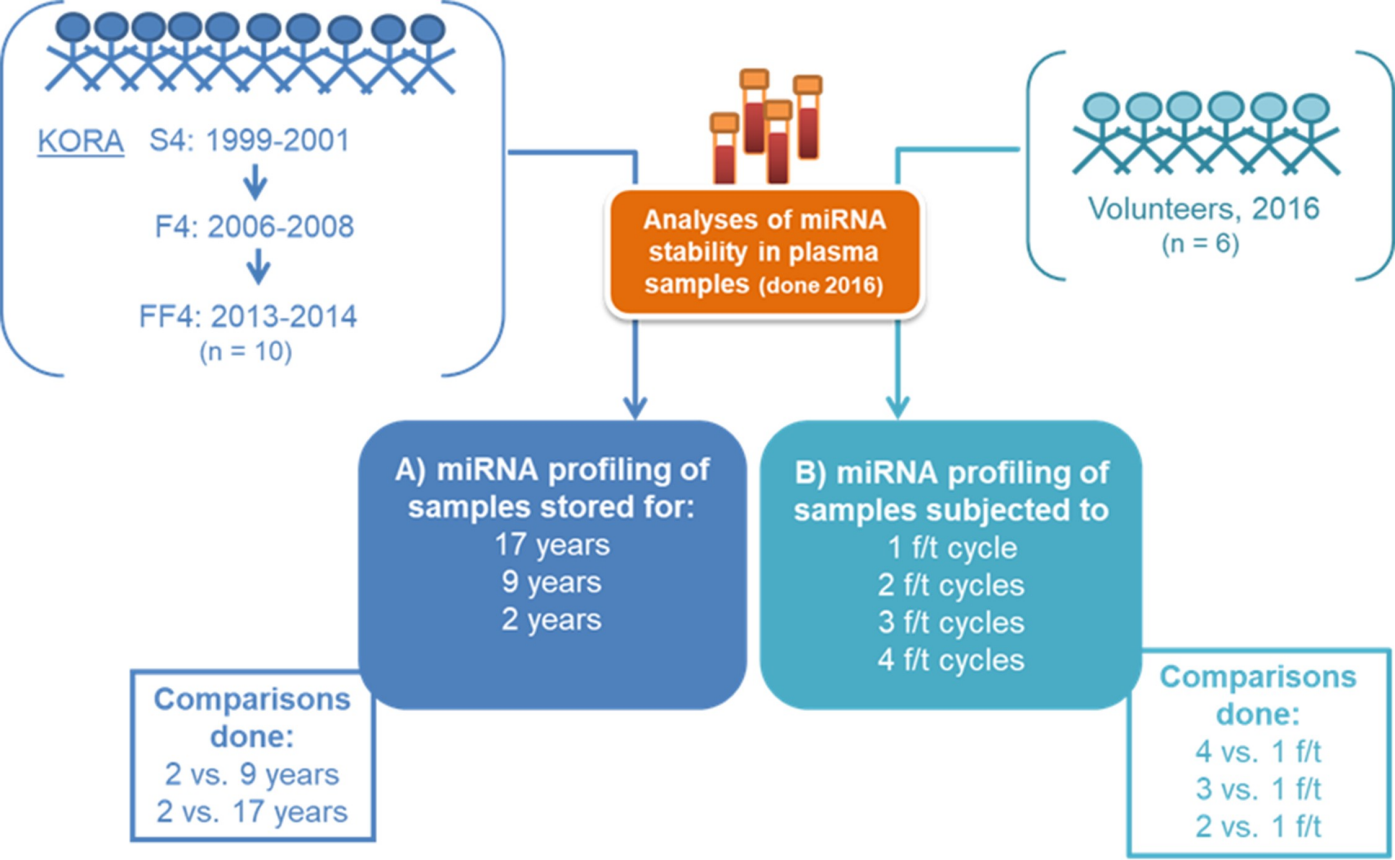
Experimental study design. (A) KORA samples from the same ten participants from three surveys were investigated for long-term storage effects on miRNA levels. (B) Plasma samples from six volunteers were analyzed for freeze-thaw influence on miRNA levels.

For the experiment on long-term storage, a total of ten participants meeting the inclusion criteria for sample selection (non-smoking, non-diabetic status, female gender, aged 25–30 years at the time of the KORA S4 study, with availability of EDTA plasma samples at all three time points) and being participants of all three surveys were identified. Plasma samples stored at -180°C (vapor phase of nitrogen) from three time points (KORA S4, F4 and FF4 surveys) were analyzed for the influence of long-term storage (2–17 years) on their circulating miRNA profile (Fig 1A).

To assess the impact of freeze-thaw (f/t) cycles on circulating miRNA profiles, and in order to avoid the unnecessary use of KORA samples for purposes verifiable also in independent samples, EDTA blood from six volunteers meeting the selection criteria as previously described was used. Samples were obtained in 2016 and processed into plasma according to the KORA study manual and the manufacturer’s instructions along with the aforementioned KORA samples (Fig 1B). Plasma samples were aliquoted into vials and stored for four weeks at -80°C until analyses; samples were not snap-frozen prior to storage at -80°C, partly due to logistic constraints and to emulate common sample handling practices. The samples were then subjected up to four freeze-thaw cycles (one cycle consisted of sample thawing on ice followed by miRNA extraction and profiling, finalizing with snap-freezing in liquid nitrogen); this number of freeze-thawing cycles was deemed to be appropriate in view of the available plasma volume and common sample handling practices. All laboratory measurements were done in 2016, and statistical analyses were conducted in 2018/2019.

### Quantitative real-time PCR (qPCR)

Circulating miRNAs were extracted from 200 μl EDTA-plasma using the miRCURY^TM^ RNA Isolation Kit–Biofluids (Exiqon A/S, Vedbaek, Denmark), adding 1 μg MS2 carrier RNA to each sample prior to RNA extraction to ensure high RNA yield and reproducibility. Synthesis of complementary DNA (cDNA) was performed using the Universal cDNA Synthesis Kit II (Exiqon A/S), whereby RNA input was optimized to 4 μl of total RNA per 10 μl reaction volume as template in the cDNA synthesis reaction. Synthetic oligonucleotides were added to the plasma samples using the miRCURY^TM^ RNA Spike-in Kit (Exiqon A/S) according to the manufacturer’s protocol to monitor cDNA synthesis and PCR amplification.

The selection of the specific set of miRNAs included in these assays was guided by their usefulness as biomarkers for sample quality, their stable expression in plasma, and their independence from status disease. The miRCURY^TM^ LNA (Exiqon A/S) miRNA profiling technology was used for two purposes: firstly to assess the robustness and quality of the RNA isolation, cDNA synthesis and overall sample quality, and secondly, to include miRNAs of interest in epidemiological research. A total of nine miRNAs were included in two qPCR panels; the full nomenclature, accession numbers and sequences for the nine miRNAs were retrieved from miRbase, release 22 [5] and are shown in S1 Table. The first qPCR panel used was the QC PCR Panel V4.M; sample quality was assessed by examining adding synthetic oligonucleotides, as well as six miRNAs expected to be highly abundant in specific sets of samples: miR-30c-5p, expressed in cerebrospinal fluid; miR-103a-3p and miR-191-5p, expressed in most tissues; miR-124-3p, expressed in kidney and urine samples; and miR-451a and miR-23a-3p, indicators of hemolysis and internal controls that are well detected in plasma and serum [35, 39]. The second qPCR panel featured four miRNAs (miR-93-5p, miR-24-3p, miR-23a-3p, and miR-33b-5p); the first three miRNAs of this panel were included in this experiment because of their stable expression in plasma samples [40–42], whereas miR-33b-5p is of epidemiological interest in the KORA survey because the methylation level of its encoding region has been found to be associated with lipid levels [43].

All qPCR assays were conducted using the Applied Biosystems 7900HT Fast Real-Time PCR System (Applied Biosystems, Carlsbad, CA, USA) following the specific recommendations for ABI instruments and the manufacturer’s instructions for each qPCR panel, e.g. number of amplification cycles (45 for both panels in order to maximize comparability between them)Data was processed using the SDS 4.2 software (Applied Biosystems). This miRNA profiling platform is sensitive and specific, as shown by the appropriate assay-specific melting temperatures in the melting curve analyses from our data (S1 Fig) and the platform’s performance in comparison to other detection systems in terms of assay cross-reactivity and specificity [24]. For all analyses in this study, miRNA expression was quantified as Cq, the PCR cycle at which the target is detected, as defined by the Minimum Information for Publication of Quantitative Real-Time PCR Experiments (MIQE) guidelines [44].

### Data preprocessing

Raw data was exported using SDS 2.4 (Applied Biosystems), and further processed using R version 3.5.3 (http://www.R-project.org) [45]. An inter-plate calibrator (IPC), UniSp3, was used to examine inter-plate technical variation in PCR amplification [23]. The detection cut-off was set at Cq > 37 and Cq values larger than this were set as missing [24].

Hemolysis was assessed by calculating the difference in expression levels (ΔCq_hemolysis_) between miR-451a, a miRNA highly expressed in red blood cells [46, 47], and miR-23a-3p, a miRNA unaffected by hemolysis and stably detected in plasma. As suggested by the manufacturer, samples with ΔCq_hemolysis_ > 7 were to be excluded from further analysis; however, none of the samples surpassed the threshold and therefore none were excluded at this point.

RNA isolation efficiency and presence of enzymatic inhibitors during both the cDNA synthesis and amplification were assessed using the synthetic oligonucleotides (UniSp2, UniSp4, UniSp5 and UniSp6). Their Cq values were tested for outliers with Grubbs’ test [48]; if an outlier was identified, the sample’s Cq distribution of the synthetic oligonucleotides was examined. qPCR data was normalized following the ΔCq method [49, 50]. The median of UniSp6 was used as a normalization factor to control for sample specific effects on assay performance and to calculate relative quantification levels of miRNAs. The choice of normalization factor was based on the availability of a common molecule in both panels, and its suitability as a reference for data analysis [21, 23, 51–53].

MiR-23a-3p was measured in both panels; the measurement included in the main statistical analysis was that from the personalized panel, whereas the measurement from the QC panel was used to assess hemolysis. MiR-124-3p was excluded from further analysis because of its low call rate (<50% of the samples) in both experiments A and B, leaving a total of eight miRNAs to be studied.

For the experiment on long-term storage (from here on referred to as experiment A), plasma samples from ten KORA participants across the KORA surveys were profiled, such that data from 10 samples and 3 time points were available for analysis for all eight miRNAs, except miR-33b-5p. This miRNA was not detected at a reliable level (Cq < 37) in a total of six samples, which were excluded from analysis. No samples were excluded in the experiment dealing with freeze-thaw cycles (hereinafter referred to as experiment B).

### Statistical analysis

Principal component analysis (PCA) was performed to monitor for batch-specific effects in the miRNA dataset. The overall effects of storage time and freeze-thaw cycles on the miRNA profiling levels were examined with one-way repeated measures analysis of variance (rANOVA) using the *Anova()* function from the R *car* v3.0 package. ΔCq data was tested for sphericity with the Mauchley test, and the Greenhouse Geisser correction was applied if this assumption was violated. For miRNAs with significant ANOVA F-ratios, Tukey’s test was used as a post-hoc analysis to determine which specific groups differed using the package *lme4* v.1.1–21. Statistical significance was considered as p < 0.05.

Considering that the sample size for both experiments was predetermined by study constraints (largest sample sizes available meeting our inclusion criteria for experiment A and attending feasibility limitations for experiment B), a sensitivity power analysis was performed using the function *wp*.*rmanova* from the R package *WebPower* v0.5 [54] to determine the minimal detectable effect with our sample in terms of Cohen’s f standardized statistic [55].

Our storage time experiment included samples from participants of a longitudinal study, which could lead to the effects of biological aging from the participants masking that of storage time on plasma miRNA levels. In order to test for the influence of biological age on miRNA levels, an independent sample from 300 participants from the KORA F4 survey was used to analyze the association between miRNA levels and biological aging; plasma samples and miRNA profiling was done following the same workflow and pipeline as aforementioned. Linear regression models were run with miRNA levels as the dependent variable; two different models were used: model 1, with age, sex, and BMI as predictor variables; and model 2, adding to those variables from model 1 technical variables (UniSp2 + ΔUniSp4—UniSp2) and blood parameters (hematocrit levels, Hct; platelet count; mean platelet volume, MPV) [56].

## Results

In this study, we investigated the influence of two pre-analytical factors, namely long-term storage at ultra-low temperatures (2, 9, and 17 years) in experiment A and repeated freeze-thaw cycles (1 to 4) in experiment B (Fig 1), on the detection of eight plasma circulating miRNAs.

### Long-term storage

For the purpose of determining the influence of storage time on circulating miRNA levels, plasma samples from ten participants and three time points were analyzed, each corresponding to a specific storage time (17, 9 and 2 years).

The power analysis showed that the minimal detectable effect in our study, with 80% power and a significance level of 0.05, was a standardized difference of means of 0.58 (Cohen’s f statistic), which is considered to be a large effect [55].

**Fig 2:**
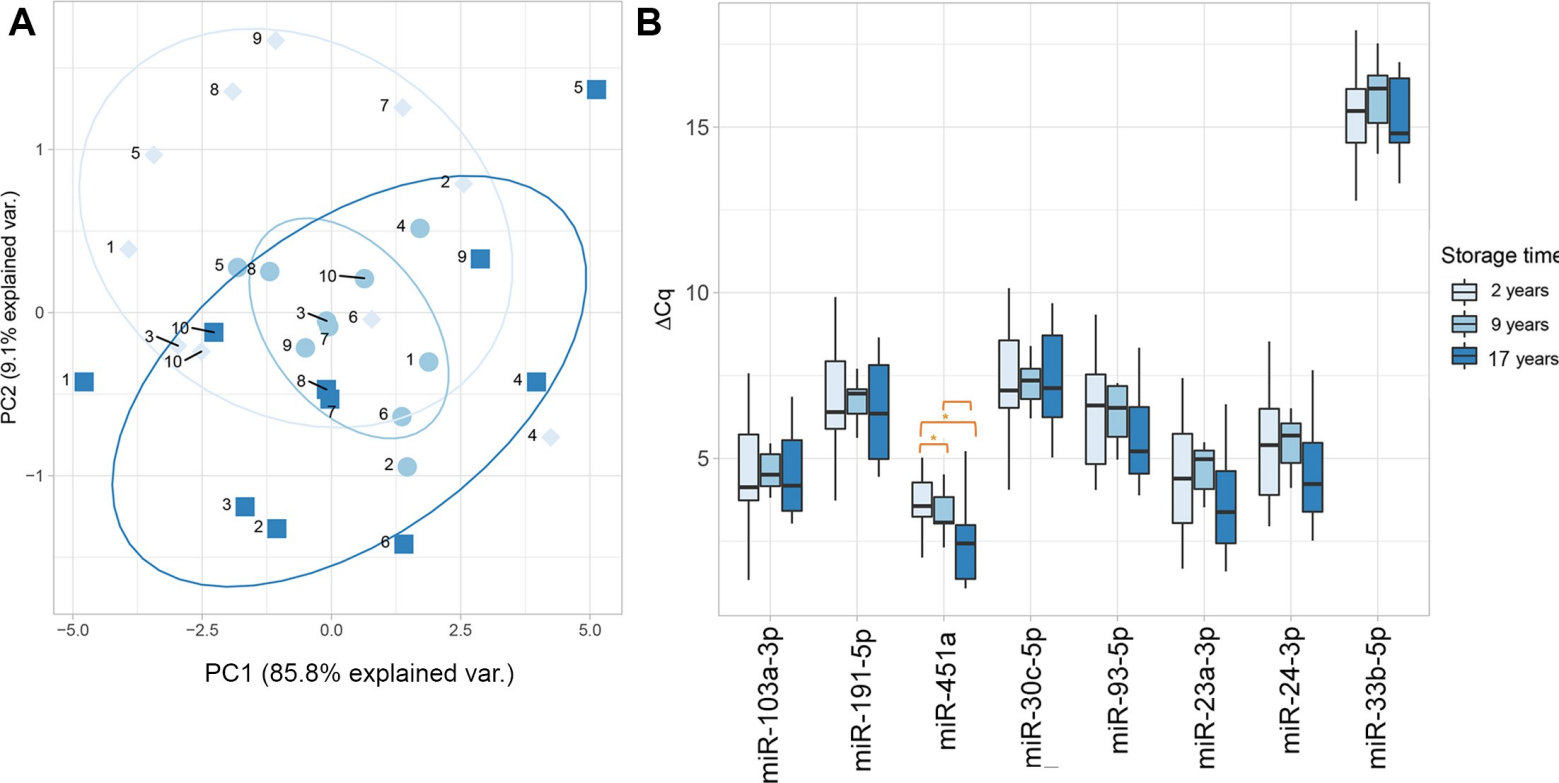
Effect of storage time on the expression levels of circulating miRNAs in plasma. **Storage duration was one of 17 years, 9 years or 2 years.** (A) Scatterplot of the first two principal component scores from the PCA analysis of storage time based on the data of the seven robustly measured miRNAs from the full sample (N_storage_ = 10). Principal components one (shown on the x-axis) and two (y-axis) explain 85.80% and 9.10% of the data variance, respectively. Symbols and colors correspond to storage time to facilitate visual inspection, and a confidence ellipse for the population mean was drawn around the grouped observations in order to visualize the variance (data with higher variance creates larger confidence ellipses). (B) Boxplots of miRNA levels based on the data from the full sample (N_storage_ = 10) for seven miRNAs and on a subset of the data with complete longitudinal observations for miR-33b-5p (N _miR-33b-5p_ = 4). The lower and upper hinges of the box present the first and third quartiles (25^th^ and 75^th^ percentiles), and the whiskers extend to 1.5 * IQR (interquartile range) in both directions; data beyond the whiskers are plotted individually. The x-axis lists the name of the miRNAs, while the y-axis shows the miRNA quantification level, calculated as ΔCq normalized to the median level of the synthetic oligonucleotide UniSp6. Smaller ΔCq values denote greater miRNA levels and are characteristic of miRNAs commonly found in plasma, whereas larger ΔCq values are characteristic of miRNAs detected in low concentrations in the sample. MiRNA measurements corresponding to 17 storage years are shown in dark blue, those corresponding to 9 years are shown in blue, and those to 2 storage years in light blue. Pairwise comparisons are shown and significant differences as determined by Tukey’s test marked with a star.

PCA, performed as an exploratory analysis, suggested some clustering regarding time along the second PC (Fig 2A), while no clustering according to individual samples was evident (S2 Fig); ΔCq distributions for all the miRNAs are shown in Fig 2B.

Table 1 displays the means and standard deviations of the quantification levels of the eight studied miRNAs, as well as the F value and p-value from the rANOVA. The overall effect of storage time was significant for miR-451a (F(2,18) = 6.82; p-value = 6.20E-03); post-hoc comparisons using Tukey’s test identified the differences to be in the comparisons of samples stored for 17 years to those stored for 9 years (difference between mean miR-451a levels from both timepoints = -0.84, SE = 0.34, p-value = 3.79E-02) and in the samples stored for 17 years compared to those stored for 2 years (difference = -1.24, SE = 0.34, p-value < 1E-03) (Fig 2B). No differences were found when comparing samples stored for 9 years to samples stored for 2 years (difference = -0.40, SE = 0.34, p-value = 0.47).

**Table 1:** Mean miRNA levels and rANOVA results from miRNA quantification levels from storage time experiment.

Condition	miR-103a-3p	miR-191-5p	miR-451a	miR-30c-5p	miR-93-5p	miR-23a-3p	miR-24-3p	miR-33b-5p ◊
2 storage years mean (SD)	4.63 (1.85)	6.83 (1.84)	3.7 (0.94)	7.47 (1.86)	6.35 (1.82)	4.36 (1.79)	5.33 (1.77)	15.39 (1.66)
9 storage years mean (SD)	4.59 (0.59)	6.78 (0.69)	3.3 (0.71)	7.34 (0.73)	6.4 (0.88)	4.7 (0.72)	5.49 (0.8)	15.92 (1.22)
17 storage years mean (SD)	4.56 (1.4)	6.43 (1.61)	2.46 (1.29)	7.33 (1.65)	5.66 (1.52)	3.7 (1.62)	4.59 (1.66)	15.3 (1.35)
rANOVA F value (p-value)	0.009 (0.99)	0.25 (0.78)	6.82 (6.2E-03)*	0.03 (0.97)	0.85 (0.44)	1.39 (0.27)	1.07 (0.36)	0.60 (0.58)

Mean and (SD) of UniSp6-normalized levels (ΔCq) of circulating miRNAs. Smaller ΔCq values denote greater miRNA levels and are characteristic of miRNAs commonly found in plasma, whereas larger ΔCq values are characteristic of miRNAs detected in low concentrations in the sample. The bottom row shows the results from the repeated measures rANOVA used to analyze the overall effect of storage time on the normalized levels of eight miRNAs (N_storage_ = 10 samples for all of the miRNAs but miR-33b-5p, see symbols below) using 2 degrees of freedom (df) for the factor (storage time) term and 18 df for the error term.

* represents statistical significance at p < 0.05

◊ Calculations for miR-33b-5p were performed on a subset with complete cases (N _miR-33b-5p_ = 4), therefore using 2 df for the factor (storage time) and 6 df for the error term

The current experimental design, in which we analyze plasma samples from the same individuals at different time points, does not allow for discrimination between the storage time effect and potential effects of other confounding variables on the miRNA levels; of these potential confounders, changes due to biological aging could be directly masking the effect of storage time (or vice versa). As an additional objective, and in order to test for the influence of biological effects of aging on miRNA levels, multiple linear regression models using miRNA levels as the outcome and independent variables (biological age, sex and BMI), blood cell parameters (haematocrit, platelet count, and mean platelet volume) and technical covariates (UniSp2, ΔUniSp4-UniSp2) [56], were run with data from an independent, larger sample of 300 participants from the KORA F4 survey. No association between the biological age (age at time of the KORA F4 survey), sex or BMI of the participants and the expression levels of the miRNAs were found in the estimated models (S2 Table and S3 Fig).

### Repeated freeze-thaw cycles

To explore the effect of freeze-thaw (f/t) cycles on miRNA detection in experiment B, plasma samples were taken from six individuals and subjected to freeze-thawing up to four times, performing miRNA profiling after each f/t cycle.

The power analysis showed that the minimal detectable effect in our study, with 80% power and a significance level of 0.05, was a standardized difference of means of 0.76 (Cohen’s f statistic), which is considered to be a large effect [55].

**Fig 3:**
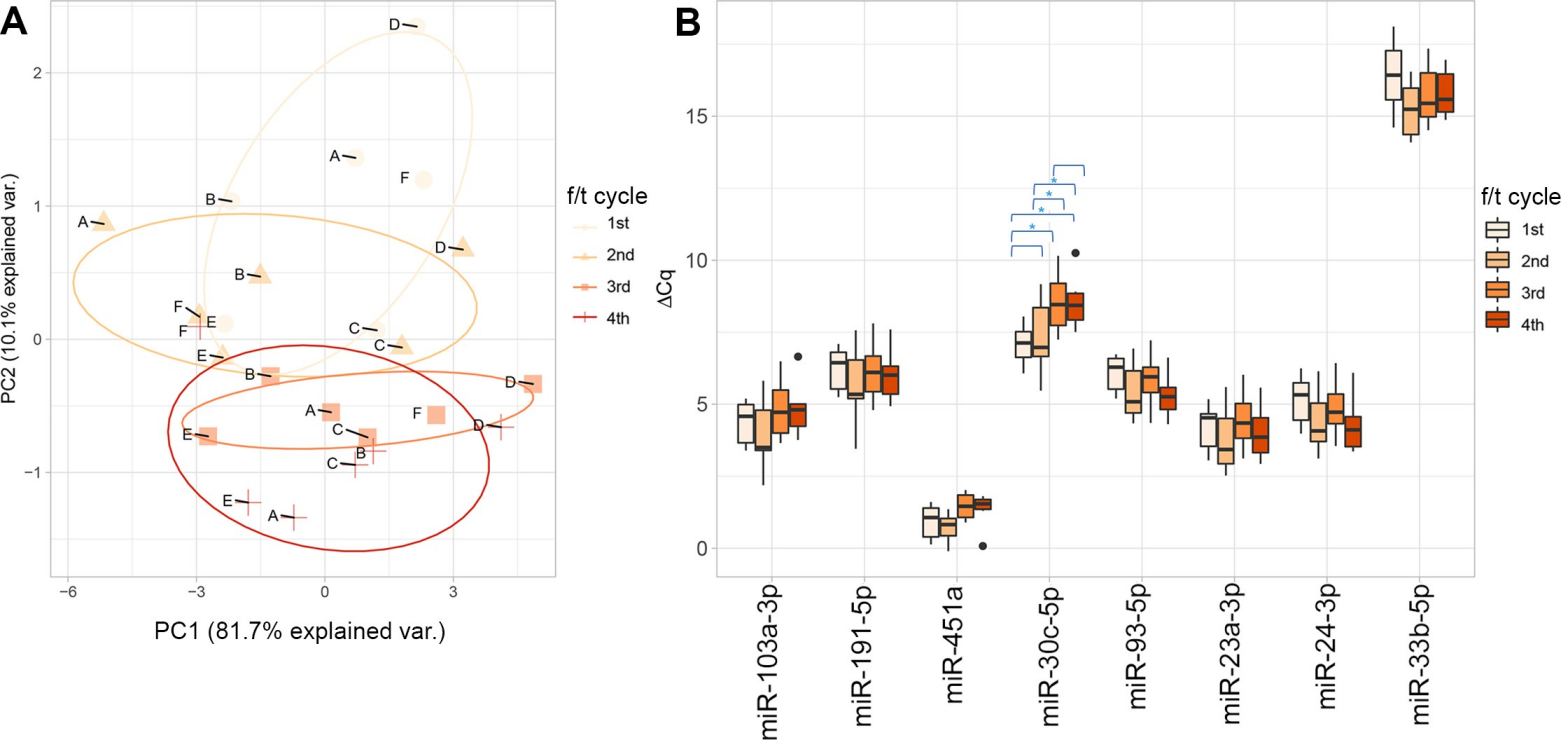
Effects of freeze-thaw cycles on detected levels of circulating miRNA in plasma samples. (A) Scatterplot of the first two principal component scores from the PCA analysis of storage time data (N_f/t_ = 6). Principal components one (shown on the x-axis) and two (y-axis) explain 81.70% and 10.10% of the data variance, respectively. Symbols and colors correspond to storage time to facilitate visual inspection, and a confidence ellipse for the population mean was drawn around the grouped observations to visualize the variance (data with higher variance creates larger confidence ellipses). (B) Boxplots of miRNA levels. The lower and upper hinges of the box present the first and third quartiles (25^th^ and 75^th^ percentiles), and the whiskers extend to 1.5 * IQR (interquartile range) in both directions; data beyond the whiskers are plotted individually. The x-axis lists the name of the miRNAs, while the y-axis shows the miRNA level, calculated as ΔCq normalized to the median level of the synthetic oligonucleotide UniSp6. Smaller ΔCq values denote greater miRNA levels and are characteristic of miRNAs commonly found in plasma, whereas larger ΔCq values are characteristic of miRNAs detected in low concentrations in the sample. Freeze-thaw (f/t) cycles are denoted by specific colors: one f/t cycle with orange, two f/t cycles with green, three f/t cycles with blue, and four f/t cycles with purple. Pair-wise comparisons are also shown, where significant differences as found by Tukey’s test are marked with a star.

By plotting the two first principal components, the PCA for this dataset suggested some clustering according to f/t cycle rather than around individuals (Fig 3A and S4 Fig); ΔCq distributions for all the miRNAs are shown in Fig 3B.

Table 2 displays the means and standard deviations of the quantification levels of the eight studied miRNAs, as well as the F value and p-value from the rANOVA. An overall effect of freeze-thawing was detected in the levels of miR-30c-5p (F(3,18) = 10.559; p-value = 5.50E-04). Differences in the level of this miRNA were observed when comparing the 3^rd^ and 4^th^ f/t cycles vs the 1^st^ f/t (Tukey’s p-values < 1E-03), as well as the 3^rd^ and 4^th^ f/t cycles vs 2^nd^ f/t cycle (p-values = 1.90E-03 and 1.54E-03, respectively) (Fig 3B). No differences were found when comparing the 2^nd^ cycle to the baseline (p-value = 0.89) nor the 4^th^ f/t cycle to the 3^rd^ f/t cycle (p-value = 0.99).

**Table 2:** Mean levels and rANOVA results from miRNA quantification levels from freeze-thawing experiment.

Condition	miR-103a-3p	miR-191-5p	miR-451a	miR-30c-5p	miR-93-5p	miR-23a-3p	miR-24-3p	miR-33b-5p
1 f/t cycle mean (SD)	4.37 (0.80)	6.23 (0.79)	0.92 (0.63)	7.08 (0.73)	6.08 (0.67)	4.2 (0.86)	5.15 (0.91)	16.4 (1.30)
2 f/t cycles mean (SD)	3.93 (1.33)	5.63 (1.45)	0.72 (0.53)	7.33 (1.39)	5.42 (1.04)	3.77 (1.20)	4.39 (1.13)	15.24 (1.02)
3 f/t cycles mean (SD)	4.85 (1.09)	6.15 (1.08)	1.46 (0.47)	8.54 (1.11)	5.85 (0.98)	4.46 (1.04)	4.86 (1.01)	15.74 (1.11)
4 f/t cycles mean (SD)	4.86 (1.01)	6.02 (0.95)	1.33 (0.64)	8.56 (0.98)	5.3 (0.81)	4.02 (0.99)	4.29 (1.02)	15.79 (0.86)
rANOVA F value (p-value)	2.36 (0.09)	0.83 (0.50)	1.88 (0.18)	10.56 (5.50E-04) *	2.16 (0.14)	1.18 (0.35)	2.97 (0.07)	1.60 (0.23)

Mean and (SD) of UniSp6-normalized levels (ΔCq) of circulating miRNAs. Smaller ΔCq values denote greater miRNA levels and are characteristic of miRNAs commonly found in plasma, whereas larger ΔCq values are characteristic of miRNAs detected in low concentrations in the sample. The bottom row shows the results from the repeated measures rANOVA used to analyze the overall effect of storage time on the normalized levels of eight miRNAs using 3 df for the factor (f/t cycle) term and 15 df for the error term.

* represents statistical significance at p < 0.05.

## Discussion

With the growing interest of biomedical research and translational medicine in circulating miRNAs as disease biomarkers and the rising reliance on biobanking in sample acquisition, research on pre-analytical factors influencing miRNA profiling is increasingly important. The abundance of circulating miRNAs is sensitive to a number of pre-analytical factors [57]; amongst such factors, contamination from cellular carryover and rupture of erythrocytes during sample processing can significantly alter the miRNA profile, as can the choice of method used for cell-free miRNA isolation and profiling [52]. Other relevant factors for miRNA expression profiling results are the storage conditions and sample handling [58]; eliminating the bias potentially introduced in these stages can be seen as critical for obtaining usable data. To investigate whether and to what extent miRNA expression profiles are biased by freeze-thawing and long-term storage, we examined the influence of up to 17 years of storage of plasma samples at ultra-low temperature (N_storage_ = 10) and the effect of multiple freeze-thaw cycles (N_f/t_ = 6) on eight circulating miRNAs.

### Long-term storage

PCA suggested some clustering in relation to time (either based on storage time, biological age of the participants or different environmental influences at different time points) (Fig 2A). In order to disentangle the potentially correlated relationship between storage time and biological differences related to aging, linear regression models were used with cross-sectional data from KORA F4. The results from this supporting analysis show no association between the levels of the eight studied miRNAs and age, sex or BMI (S2 Table); these findings are also in line with those from a previous study of circulating miRNAs in a similar population [56]. Moreover, PCA did not suggest clustering in terms of inter-individual variability (S2 Fig), in contrast to a previous report [7]. The clustering along PC2 could be due to unmeasured variables unrelated to storage time, but related to conditions at the time of initial storage, which could include varying degrees of cell contamination [59], as suggested by the results obtained from the analysis of individual miRNA levels.

According to the rANOVA analysis, long-term storage up to 17 years at -180°C (vapor phase of liquid nitrogen) showed no large effect on the levels of miR-30c-5p, miR-103a-3p or miR-191-5p, all of which are included in the manufacturer’s Plasma/Serum QC Panel (Exiqon). This underlines the suitability of these miRNAs as reference miRNAs to control for sample quality prior to large-scale miRNA profiling, and provides additional evidence, beyond that offered by extensive profiling, of their robustness to biological effects [23]. No large effects of storage duration on the levels of miR-23a-3p, miR-93-5p, miR-24-3p or miR-33b-5p were observed.

However, results from the rANOVA initially suggested an overall storage effect on the miR-451a levels in plasma samples, which were elevated when stored for 17 years compared to those stored for two and nine years (Fig 2B). Nevertheless, given that miR-451a is a well-known marker for hemolysis and is highly abundant in mature myeloid and red blood cells [36], potential contamination of plasma with cellular material and miRNAs from apoptotic or lysed cells (e.g. red blood cells or platelets) could explain this difference [60, 61]. The higher level of miR-451a identified in older samples compared to more recent samples could to be an indicator of a relatively greater degree of hemolysis and contamination with cellular material at the time of processing [33, 59]. This could be explained by a few differences at the time of sample collection during the KORA S4 survey, such as stress conditions imposed by blood drawing conducted using slightly narrower cannulas or by freezing the samples for up to eleven days at -25°C prior to long-term freezing (KORA manuals, not public). However, this difference in detected levels is not considered large enough to indicate unreliability of the miRNA profiling in the older samples, as no samples failed the hemolysis threshold recommended by the manufacturer (ΔCq_hemolysis_ > 7).

In general, our findings are in line with several other studies examining miRNA-specific effects of storage time. A number of studies have shown that stability is related to the type of transport mechanism and encapsulation characteristic of every miRNA [53, 62]. On the one hand, miR-24-3p and miR-451a, existing as free miRNAs and packed in exosomes, have been previously reported to be largely unaffected by storage conditions [62]. Another study showed that whereas total miRNA yield decreased after 8 years of storage at -80°C, miR-30c-5p is protected from degradation in serum samples frozen for up to 14 years as it is often encapsulated in extracellular vesicles [53]. On the other hand, a recent study found that storage of samples at -80°C for up to nine months affected miRNA stability in whole blood but not in plasma [57], evidence pointing to the stability of cell-free miRNA fractions regardless of their specific transport mechanisms. It is not clear whether the other four miRNAs analyzed here are freely circulating, transported in extracellular vesicles or if they form complexes with other molecules, and so their results offer no hints on the role of transport mechanism in miRNA stability.

Another factor influencing miRNA stability through time is the type of sample. Preprocessed blood samples (e.g. plasma or serum) are to be preferred to whole blood samples for long-term storage [57]. Presence of platelet derived miRNAs in serum suggests that the coagulation process may affect the spectrum of extracellular miRNA in blood [22], thus favoring plasma as the optimal sample for circulating miRNA assessment. This further highlights the importance of proper pre-analytical sample processing in order to avoid cellular contamination and minimize technical variations in miRNA profiling.

A study of similar design to ours (examining miRNAs by means of qPCR, using plasma samples collected in a longitudinal manner and stored in a biobank at -80° C) observed sequence-specific miRNA degradation patterns after 14 years of storage, with lower miRNA levels linked to a higher number of AU or UA dinucleotide repetitions in the miRNA’s sequence [32]. The miRNAs analyzed in our study featured one to three of these dinucleotide repetitions in their sequences (S1 Table) and did not show any sequence-specific degradation patterns; the aforementioned study analyzed miRNAs whose sequences had up to five of such dinucleotide repetitions, so this difference in the number of repetitions may explain why our results do not seem to support the theory of miRNA instability being dependent on the number of AU sequences.

Our study shows no statistically significant evidence for large effects for long-term storage conducted at ultra-low temperatures on levels of a selected set of eight miRNAs. Altogether, our results offer specific evidence from a set of miRNAs supporting the conclusions presented by Hebels and colleagues, who did not find effects of long-term storage (13–17 years) in proteomics, metabolomics, transcriptomics or DNA methylation from biobank blood samples [63].

### Repeated freeze-thaw cycles

Some clustering could be observed in the PCA regarding f/t cycles (Fig 3A) but not regarding variability within individuals (S4 Fig), in contrast to a previous report [7]. According to the rANOVA analysis, up to four freeze-thawing cycles did not have a large effect on the levels of seven of the miRNAs studied. An overall, large freeze-thawing effect was observed only for miR-30c-5p, which was detected at significantly lower levels after three and four sequential f/t cycles (Table 2 and Fig 3B). Lastly, a borderline significant effect was observed for miR-24-3p (p-value = 0.06).

The observed effect on miR-30c-5p might be explained in terms of the stability conferred to circulating miRNAs by its molecular interactions, either via complex formation with other molecules or vesiculation (process in which miRNAs are packed into vesicles). Evidence for the protective effect of molecular interactions in miRNA stability has been observed in multiple studies [28, 53, 57, 64].

Of the eight miRNAs studied here, miR-24-3p has been examined in a larger number of studies. An early study in the field of circulating miRNAs observed no effects of up to eight f/t cycles on the plasma levels of this miRNA, results suggesting its presence as a form resistant to RNase activity, and hence its existence within secreted exosomes [28]. Another study later confirmed and extended these findings using a larger sample size and serum samples subjected to up to four f/t cycles [65]. The effect of freeze-thawing has also been studied on miR-451a, a miRNA existing both within and outside microvesicles (MV) [19]. In line with our results, no significant effects were found in a smaller sample size from biobank serum samples [53]; however, a later study reported a significant decrease of total miRNA levels in plasma after two f/t cycles but no differences in exosomal miRNA levels [62]. Another factor explaining our results could be platelet contamination in plasma samples [21, 66]. While plasma with a low amount of platelets has been observed to be insensitive to freeze-thawing, miRNA levels in platelet rich plasma do not change with additional freeze-thawing cycles once platelet damage has occurred; moreover, platelets are more sensitive to external conditions than other miRNA carriers like extracellular vesicles [66]. The seemingly lower levels of miR-451a in f/t cycle 3 and 4 (although not statistically significant), as well as those of miR-30c-5p (Fig 3B), could suggest some degree of residual platelet contamination. This could explain the slightly decreased levels of miRNA after the third f/t cycle, the point where platelet damage possibly occurred and beyond which miRNA levels do not decrease further. Alternatively, the suggestive pattern displayed by miR-24-3p, whose levels seemed to increase after 4 f/t cycles (p-value = 0.06) (Fig 3B), could be due to increasing separation of miRNAs from their binding proteins with an increasing number of f/t cycles, allowing improved detection via RT-qPCR, in line with the observations made by a previous study [67].

It has not yet been described whether the eight studied miRNAs are present in extracellular vesicles or bound to protein complexes (such as Ago2 or HDL); however, 90% of total extracellular miRNA have been identified as not being vesicle-associated circulating miRNAs [18], which increases the likelihood of this set of miRNAs belonging to the latter group. Furthermore, most of the miRNAs studied here have been detected in both cell-free and cellular fractions (also considered as the “contaminating” fraction composed by white blood cells, red blood cells and leukocytes) [19], which additionally underlines the potential influence of sample processing and residual platelets in miRNA profiling.

Our study demonstrates that the selected eight miRNAs can be stably detected in samples stored at ultra-low temperatures for long storage periods, as well as a robustness of all miRNAs studied here but miR-30c-5p to freeze-thawing cycles. Our findings offer insights into the behavior of a set of miRNAs in plasma samples processed and stored according to biobank protocols, thus being of great interest to research on circulating miRNAs and to studies conducted using biobank samples.

### Strengths

Numerous previous studies have examined the effects of pre-analytical variables, yet little research has been done with actual samples from serial visits from a longitudinal, population-based study. Our 17 years of storage represents, to the best of our knowledge, the longest storage period among the reported studies dealing with miRNA expression from plasma biobank samples. Moreover, we were able to use the follow-up samples from ten participants over this time period in a longitudinal cohort, which constitutes a unique study design. Additionally, we were able to complement this approach with a supplementary analysis from a larger sample examining the association between the studied miRNAs and biological aging in order to disentangle the potentially correlated relationship between storage time and biological differences related to aging. The further adjustment for platelet levels in this model allowed us to examine the association between miRNA levels and age, sex and BMI, independently from potential residual platelet contamination.

### Limitations

In this study, we examined eight miRNAs, so this short list by no means captures the variability in stability of all circulating plasma miRNAs, but only reflects the effects of storage and freeze-thawing on this set of reference miRNAs. Our findings should be interpreted considering study-specific settings (namely the specific set of miRNAs included, sample type, storage duration and temperature) in further comparisons. Sample size is also a limitation since our study design only allowed for the detection of large and very large effects, thus the studied pre-analytical factors could have smaller effects on miRNA detection levels that would go unnoticed by our study design as false-negative results. Additionally, our comparisons were always performed with respect to plasma samples that had been already subjected to one f/t cycle because we did not profile circulating miRNAs from fresh, newly extracted blood samples; however, this situation better represents the day-to-day dynamics of biobanks and research centers, where tissue and blood samples are stored after their collection and analyzed at some point in the future. Finally, regarding the choice of normalization factor, we did not use a stable endogenous miRNA in the data normalization and opted for using a synthetic oligonucleotide instead. However, since the encapsulation and transport mechanism are miRNA-specific characteristics that could interfere with detection of the miRNA, by utilizing a synthetic oligonucleotide we prevented such confounding and achieved comparability between our two qPCR panels.

## Conclusion

Our study intended to share our experience with two important pre-analytical variables in the profiling of a set of eight miRNAs in plasma samples stored and/or processed following biobanking protocols, investigating the influence of up to 17 years of long-term storage at ultra-low temperatures and multiple freeze-thaw cycles on miRNA biomarkers. We found no large effects of storage at ultra-low temperatures (e.g. in vapor phase of liquid nitrogen) on the levels of a set of eight miRNAs in plasma samples stored for decades. We also identified resistance to multiple freeze-thaw cycles in six of the eight plasma miRNAs, whereas miR-30c-5p showed a very large effect and miR-24-3p to a lesser extent. The results presented here thus support the potential use of a subset of the miRNAs studied here, namely miR-103a-3p, miR-191-5p, miR-23a-3p, and miR-93-5p, as stable references in future circulating miRNA studies, given that their levels are not largely affected by storage time at ultra-low temperatures nor freeze-thawing conditions. Moreover, miR-30c-5p could also be used as a marker to detect freeze-thawing related changes in circulating miRNA levels. Our results also highlight the importance of appropriate biobanking practices in sample handling that facilitate longitudinal epidemiological research.

## Supporting information

S1 Fig Plots from melting curve analyses. Panel A shows the melting curve analysis from the miR-30c-5p assay. Panel B shows the melting curve analysis from the miR-451a assay.
(TIFF)

S2 Fig PCA of storage time data. Scatterplot of the first two principal component scores from the PCA analysis of storage time data. Principal components one (shown on the x-axis) and two (y-axis) explain 85.80% and 9.10% of the data variance, respectively. Colors correspond to the ten subjects from the KORA study to facilitate visual inspection, and a confidence ellipse for the population mean was drawn around the grouped observations in order to visualize the variance (data with higher variance creates larger confidence ellipses). Observations from the three time points deriving from the same subject are labeled with an ordinal number corresponding to the subject.
(TIFF)

S3 Fig Association of studied miRNAs with age, sex, BMI and other covariates. Dot plot with the p-value results from model 2 (miRNA level ~ age + sex + BMI + UniSp2 + ΔUniSp4—UniSp2 + Hct + platelets + MPV) as obtained from the linear regression analysis done with data from 300 participants from the KORA F4 survey. The negative logarithm of the p-value of the association between each miRNA and the covariates included in model 2 is plotted in the x-axis, while the y-axis lists the studied miRNAs. The different covariates are color and symbol coded. The results for the two technical parameters UniSp2 and Δ(UniSp4-UniSp2), corresponding to the oligonucleotides spiked-in during sample processing used as covariates in the model to adjust for technical variability [56], are not shown in this plot as to favor simplicity.
(TIFF)

S4 Fig PCA of freeze-thaw data. Scatterplot of the first two principal component scores from the PCA analysis of storage time data. Principal components one (shown on the x-axis) and two (y-axis) explain 81.70% and 10.10% of the data variance, respectively. Symbols and colors correspond to the six volunteers (subjects A to F) to facilitate visual inspection, and a confidence ellipse for the population mean was drawn around the grouped observations in order to visualize the variance (data with higher variance creates larger confidence ellipses).
(TIFF)

S1 Table MiRNA accession number, nomenclature and sequence. The full nomenclature, accession numbers and sequences for the nine miRNAs studied in this project, as retrieved from miRbase, release 22.
(DOC)

S2 Table Association of studied miRNAs with age, sex, BMI and other covariates. The table shows the results from the linear regression models used to analyze data from 300 participants from the KORA F4 study. miRNAs were profiled using the Exiqon Serum/Plasma Focus microRNA PCR Panel V3.M (Exiqon A/S) as described by the manufacturer’s protocol, and data went through the same quality control measures as described in the main Methods section. Information on BMI and the other covariates used in the models was collected at the time of interview within the KORA F4 survey. Two models were used: model 1, with age, sex, and BMI as predictor variables; and model 2, adding to those variables from model 1 technical variables (UniSp2 + ΔUniSp4—UniSp2) and blood parameters (hematocrit levels, Hct; platelet count; mean platelet volume, MPV) [56].
(DOCX)

## References

[1] Ellervik C, Vaught J. Preanalytical Variables Affecting the Integrity of Human Biospecimens in Biobanking. Clinical Chemistry. 2015;61(7):914–34. 10.1373/clinchem.2014.228783 25979952

[2] Bartel DP. MicroRNAs: genomics, biogenesis, mechanism, and function. Cell. 2004;116(2):281–97. Epub 2004/01/28. 10.1016/s0092-8674(04)00045-5 . 14744438

[3] Finnegan EF, Pasquinelli AE. MicroRNA biogenesis: regulating the regulators. Critical reviews in biochemistry and molecular biology. 2013;48(1):51–68. Epub 2012/11/21. 10.3109/10409238.2012.738643 23163351 PMC3557704

[4] Breving K, Esquela-Kerscher A. The complexities of microRNA regulation: mirandering around the rules. The international journal of biochemistry & cell biology. 2010;42(8):1316–29. Epub 2009/10/06. 10.1016/j.biocel.2009.09.016 . 19800023

[5] Kozomara A, Birgaoanu M, Griffiths-Jones S. miRBase: from microRNA sequences to function. Nucleic Acids Research. 2018;47(D1):D155–D62. 10.1093/nar/gky1141 30423142 PMC6323917

[6] Pritchard CC, Cheng HH, Tewari M. MicroRNA profiling: approaches and considerations. Nat Rev Genet. 2012;13(5):358–69. 10.1038/nrg3198 . 22510765 PMC4517822

[7] Ammerlaan W, Betsou F. Intraindividual Temporal miRNA Variability in Serum, Plasma, and White Blood Cell Subpopulations. Biopreserv Biobank. 2016;14(5):390–7. 10.1089/bio.2015.0125 . 27096687

[8] Ambros V. The functions of animal microRNAs. Nature. 2004;431(7006):350–5. 10.1038/nature02871 . 15372042

[9] Plasterk RH. Micro RNAs in animal development. Cell. 2006;124(5):877–81. 10.1016/j.cell.2006.02.030 . 16530032

[10] Gilad S, Meiri E, Yogev Y, Benjamin S, Lebanony D, Yerushalmi N, et al Serum microRNAs are promising novel biomarkers. PLoS One. 2008;3(9):e3148 10.1371/journal.pone.0003148 18773077 PMC2519789

[11] Kuner R, Brase JC, Sultmann H, Wuttig D. microRNA biomarkers in body fluids of prostate cancer patients. Methods. 2013;59(1):132–7. 10.1016/j.ymeth.2012.05.004 . 22652624

[12] Chakraborty C, Sharma AR, Sharma G, Doss CGP, Lee S-S. Therapeutic miRNA and siRNA: Moving from Bench to Clinic as Next Generation Medicine. Molecular Therapy—Nucleic Acids. 8:132–43. 10.1016/j.omtn.2017.06.005 28918016 PMC5496203

[13] Peng F, He J, Chen Loo J, Kong SK, Li B, Gu D. IdentificationofserumMicroRNAsasdiagnosticbiomarkersforinfluenzaH7N9infection. VirologyReports. 2017;7:1–8. 10.1016/j.virep.2016.11.001.

[14] Jae-Eun L, Young-Youl K. Impact of Preanalytical Variations in Blood-Derived Biospecimens on Omics Studies: Toward Precision Biobanking? OMICS: A Journal of Integrative Biology. 2017;21(9):499–508. 10.1089/omi.2017.0109 . 28873014

[15] Moore HM, Compton CC, Alper J, Vaught JB. International Approaches to Advancing Biospecimen Science. Cancer Epidemiology Biomarkers & Prevention. 2011; 20(5):729–32. 10.1158/1055-9965.epi-11-0021 21430299 PMC3089662

[16] Simeon-Dubach D, Burt AD, Hall PA. Quality really matters: the need to improve specimen quality in biomedical research. The Journal of Pathology. 2012;228(4):431–3. 10.1002/path.4117 23023660

[17] Nair VS, Pritchard CC, Tewari M, Ioannidis JP. Design and Analysis for Studying microRNAs in Human Disease: A Primer on -Omic Technologies. American journal of epidemiology. 2014;180(2):140–52. Epub 2014/06/27. 10.1093/aje/kwu135 24966218 PMC4082346

[18] Arroyo JD, Chevillet JR, Kroh EM, Ruf IK, Pritchard CC, Gibson DF, et al Argonaute2 complexes carry a population of circulating microRNAs independent of vesicles in human plasma. Proceedings of the National Academy of Sciences of the United States of America. 2011;108(12):5003–8. Epub 2011/03/09. 10.1073/pnas.1019055108 21383194 PMC3064324

[19] Duttagupta R, Jiang R, Gollub J, Getts RC, Jones KW. Impact of cellular miRNAs on circulating miRNA biomarker signatures. PLoS One. 2011;6(6):e20769 Epub 2011/06/24. 10.1371/journal.pone.0020769 21698099 PMC3117799

[20] Pritchard CC, Kroh E, Wood B, Arroyo JD, Dougherty KJ, Miyaji MM, et al Blood cell origin of circulating microRNAs: a cautionary note for cancer biomarker studies. Cancer prevention research. 2012;5(3):492–7. 10.1158/1940-6207.CAPR-11-0370 22158052 PMC4186243

[21] McDonald JS, Milosevic D, Reddi HV, Grebe SK, Algeciras-Schimnich A. Analysis of circulating microRNA: preanalytical and analytical challenges. Clin Chem. 2011;57(6):833–40. 10.1373/clinchem.2010.157198 . 21487102

[22] Wang K, Yuan Y, Cho JH, McClarty S, Baxter D, Galas DJ. Comparing the MicroRNA spectrum between serum and plasma. PLoS One. 2012;7(7):e41561 Epub 2012/08/04. 10.1371/journal.pone.0041561 22859996 PMC3409228

[23] Blondal T, Jensby Nielsen S, Baker A, Andreasen D, Mouritzen P, Wrang Teilum M, et al Assessing sample and miRNA profile quality in serum and plasma or other biofluids. Methods. 2013;59(1):S1–6. 10.1016/j.ymeth.2012.09.015 . 23036329

[24] Mestdagh P, Hartmann N, Baeriswyl L, Andreasen D, Bernard N, Chen C, et al Evaluation of quantitative miRNA expression platforms in the microRNA quality control (miRQC) study. Nature methods. 2014;11(8):809–15. Epub 2014/06/30. 10.1038/nmeth.3014 . 24973947

[25] Sourvinou IS, Markou A, Lianidou ES. Quantification of circulating miRNAs in plasma: effect of preanalytical and analytical parameters on their isolation and stability. The Journal of molecular diagnostics: JMD. 2013;15(6):827–34. Epub 2013/08/31. 10.1016/j.jmoldx.2013.07.005 . 23988620

[26] Chen X, Ba Y, Ma L, Cai X, Yin Y, Wang K, et al Characterization of microRNAs in serum: a novel class of biomarkers for diagnosis of cancer and other diseases. Cell Research. 2008;18:997 10.1038/cr.2008.282 https://www.nature.com/articles/cr2008282#supplementary-information. 18766170

[27] Vickers KC, Palmisano BT, Shoucri BM, Shamburek RD, Remaley AT. MicroRNAs are transported in plasma and delivered to recipient cells by high-density lipoproteins. Nature Cell Biology. 2011;13:423 10.1038/ncb2210 https://www.nature.com/articles/ncb2210#supplementary-information. 21423178 PMC3074610

[28] Mitchell PS, Parkin RK, Kroh EM, Fritz BR, Wyman SK, Pogosova-Agadjanyan EL, et al Circulating microRNAs as stable blood-based markers for cancer detection. Proceedings of the National Academy of Sciences of the United States of America. 2008;105(30):10513–8. 10.1073/pnas.0804549105 18663219 PMC2492472

[29] Koberle V, Pleli T, Schmithals C, Augusto Alonso E, Haupenthal J, Bonig H, et al Differential stability of cell-free circulating microRNAs: implications for their utilization as biomarkers. PLoS One. 2013;8(9):e75184 10.1371/journal.pone.0075184 24073250 PMC3779196

[30] Cuhadar S, Koseoglu M, Atay A, Dirican A. The effect of storage time and freeze-thaw cycles on the stability of serum samples. Biochemia medica. 2013;23(1):70–7. 10.11613/BM.2013.009 . 23457767 PMC3900085

[31] Paltiel L, Ronningen KS, Meltzer HM, Baker SV, Hoppin JA. Evaluation of Freeze Thaw Cycles on stored plasma in the Biobank of the Norwegian Mother and Child Cohort Study. Cell Preserv Technol. 2008;6(3):223–30. 10.1089/cpt.2008.0012 20428472 PMC2860294

[32] Balzano F, Deiana M, Dei Giudici S, Oggiano A, Baralla A, Pasella S, et al miRNA Stability in Frozen Plasma Samples. Molecules. 2015;20(10):19030–40. Epub 2015/10/23. 10.3390/molecules201019030 26492230 PMC6331950

[33] Cheng HH, Yi HS, Kim Y, Kroh EM, Chien JW, Eaton KD, et al Plasma processing conditions substantially influence circulating microRNA biomarker levels. PLoS One. 2013;8(6):e64795 10.1371/journal.pone.0064795 23762257 PMC3676411

[34] Binderup HG, Houlind K, Madsen JS, Brasen CL. Pre-storage centrifugation conditions have significant impact on measured microRNA levels in biobanked EDTA plasma samples. Biochem Biophys Rep. 2016;7:195–200. 10.1016/j.bbrep.2016.06.005 28955906 PMC5613297

[35] Kirschner MB, Edelman JJ, Kao SC, Vallely MP, van Zandwijk N, Reid G. The Impact of Hemolysis on Cell-Free microRNA Biomarkers. Front Genet. 2013;4:94 10.3389/fgene.2013.00094 23745127 PMC3663194

[36] Grasedieck S, Scholer N, Bommer M, Niess JH, Tumani H, Rouhi A, et al Impact of serum storage conditions on microRNA stability. Leukemia. 2012;26(11):2414–6. 10.1038/leu.2012.106 . 22504138

[37] Holle R, Happich M, Lowel H, Wichmann HE, Group MKS. KORA—a research platform for population based health research. Gesundheitswesen. 2005;67 Suppl 1:S19–25. 10.1055/s-2005-858235 . 16032513

[38] World Medical A. World Medical Association Declaration of Helsinki: ethical principles for medical research involving human subjects. JAMA. 2013;310(20):2191–4. 10.1001/jama.2013.281053 . 24141714

[39] Pizzamiglio S, Zanutto S, Ciniselli CM, Belfiore A, Bottelli S, Gariboldi M, et al A methodological procedure for evaluating the impact of hemolysis on circulating microRNAs. Oncol Lett. 2017;13(1):315–20. 10.3892/ol.2016.5452 28123561 PMC5244842

[40] Andersen CL, Jensen JL, Orntoft TF. Normalization of real-time quantitative reverse transcription-PCR data: a model-based variance estimation approach to identify genes suited for normalization, applied to bladder and colon cancer data sets. Cancer research. 2004;64(15):5245–50. Epub 2004/08/04. 10.1158/0008-5472.CAN-04-0496 . 15289330

[41] Vandesompele J, De Preter K, Pattyn F, Poppe B, Van Roy N, De Paepe A, et al Accurate normalization of real-time quantitative RT-PCR data by geometric averaging of multiple internal control genes. Genome biology. 2002;3(7):RESEARCH0034. Epub 2002/08/20. 10.1186/gb-2002-3-7-research0034 12184808 PMC126239

[42] Vandesompele J, Kubista M, Pfaffl M. Reference gene validation software for improved normalization Real-Time PCR: Current Technology and Applications (Edited by: Julie Logan, Kirstin Edwards and Nick Saunders) Caister Academic Press, UK (2009) 2009.

[43] Pfeiffer L, Wahl S, Pilling LC, Reischl E, Sandling JK, Kunze S, et al DNA methylation of lipid-related genes affects blood lipid levels. Circulation Cardiovascular genetics. 2015;8(2):334–42. Epub 2015/01/15. 10.1161/CIRCGENETICS.114.000804 25583993 PMC5012424

[44] Bustin SA, Benes V, Garson JA, Hellemans J, Huggett J, Kubista M, et al The MIQE guidelines: minimum information for publication of quantitative real-time PCR experiments. Clin Chem. 2009;55(4):611–22. Epub 2009/02/28. 10.1373/clinchem.2008.112797 . 19246619

[45] Computing RFfS. R: A language and environment for statistical computing. Vienna, Austria2016.

[46] Wang K, Zhang S, Weber J, Baxter D, Galas DJ. Export of microRNAs and microRNA-protective protein by mammalian cells. Nucleic Acids Res. 2010;38(20):7248–59. Epub 2010/07/10. 10.1093/nar/gkq601 20615901 PMC2978372

[47] Williams Z, Ben-Dov IZ, Elias R, Mihailovic A, Brown M, Rosenwaks Z, et al Comprehensive profiling of circulating microRNA via small RNA sequencing of cDNA libraries reveals biomarker potential and limitations. Proceedings of the National Academy of Sciences of the United States of America. 2013;110(11):4255–60. Epub 2013/02/27. 10.1073/pnas.1214046110 23440203 PMC3600502

[48] Burns MJ, Nixon GJ, Foy CA, Harris N. Standardisation of data from real-time quantitative PCR methods—evaluation of outliers and comparison of calibration curves. BMC biotechnology. 2005;5:31 Epub 2005/12/13. 10.1186/1472-6750-5-31 16336641 PMC1326201

[49] Livak KJ, Schmittgen TD. Analysis of relative gene expression data using real-time quantitative PCR and the 2(-Delta Delta C(T)) Method. Methods. 2001;25(4):402–8. 10.1006/meth.2001.1262 . 11846609

[50] Schmittgen TD, Livak KJ. Analyzing real-time PCR data by the comparative CT method. Nature Protocols. 2008;3(6):1101–8. 10.1038/nprot.2008.73 18546601

[51] Morata-Tarifa C, Picon-Ruiz M, Griñan-Lison C, Boulaiz H, Perán M, Garcia MA, et al Validation of suitable normalizers for miR expression patterns analysis covering tumour heterogeneity. Scientific Reports. 2017;7:39782 10.1038/srep39782 https://www.nature.com/articles/srep39782#supplementary-information. 28051134 PMC5209713

[52] Kroh EM, Parkin RK, Mitchell PS, Tewari M. Analysis of circulating microRNA biomarkers in plasma and serum using quantitative reverse transcription-PCR (qRT-PCR). Methods. 2010;50(4):298–301. Epub 2010/02/12. 10.1016/j.ymeth.2010.01.032 20146939 PMC4186708

[53] Andreu Z, Rivas E, Sanguino-Pascual A, Lamana A, Marazuela M, Gonzalez-Alvaro I, et al Comparative analysis of EV isolation procedures for miRNAs detection in serum samples. Journal of extracellular vesicles. 2016;5:31655 Epub 2016/06/23. 10.3402/jev.v5.31655 27330048 PMC4916259

[54] Zhang Z, Mai Y. ‘WebPower’: An R Package for Basic and Advanced Statistical Power Analysis2018.

[55] Zhang Z, Yuan K-H. Practical Statistical Power Analysis Using Webpower and R2018.

[56] Ameling S, Kacprowski T, Chilukoti RK, Malsch C, Liebscher V, Suhre K, et al Associations of circulating plasma microRNAs with age, body mass index and sex in a population-based study. BMC medical genomics. 2015;8:61 10.1186/s12920-015-0136-7 26462558 PMC4604724

[57] Glinge C, Clauss S, Boddum K, Jabbari R, Jabbari J, Risgaard B, et al Stability of Circulating Blood-Based MicroRNAs–Pre-Analytic Methodological Considerations. PLoS ONE. 2017;12(2):e0167969 10.1371/journal.pone.0167969 PMC5289450. 28151938 PMC5289450

[58] Mraz M, Malinova K, Mayer J, Pospisilova S. MicroRNA isolation and stability in stored RNA samples. Biochem Biophys Res Commun. 2009;390(1):1–4. 10.1016/j.bbrc.2009.09.061 . 19769940

[59] Kirschner MB, Kao SC, Edelman JJ, Armstrong NJ, Vallely MP, van Zandwijk N, et al Haemolysis during sample preparation alters microRNA content of plasma. PLoS One. 2011;6(9):e24145 Epub 2011/09/13. 10.1371/journal.pone.0024145 21909417 PMC3164711

[60] Hastings ML, Palma J, Duelli DM. Sensitive PCR-based quantitation of cell-free circulating microRNAs. Methods. 2012;58(2):144–50. 10.1016/j.ymeth.2012.07.026 22884953 PMC3508311

[61] Kim DJ, Linnstaedt S, Palma J, Park JC, Ntrivalas E, Kwak-Kim JY, et al Plasma components affect accuracy of circulating cancer-related microRNA quantitation. The Journal of molecular diagnostics: JMD. 2012;14(1):71–80. 10.1016/j.jmoldx.2011.09.002 22154918 PMC3338348

[62] Ge Q, Zhou Y, Lu J, Bai Y, Xie X, Lu Z. miRNA in plasma exosome is stable under different storage conditions. Molecules. 2014;19(2):1568–75. Epub 2014/01/30. 10.3390/molecules19021568 24473213 PMC6271968

[63] Hebels DG, Georgiadis P, Keun HC, Athersuch TJ, Vineis P, Vermeulen R, et al Performance in omics analyses of blood samples in long-term storage: opportunities for the exploitation of existing biobanks in environmental health research. Environmental health perspectives. 2013;121(4):480–7. Epub 2013/02/07. 10.1289/ehp.1205657 23384616 PMC3620742

[64] Köberle V, Pleli T, Schmithals C, Augusto Alonso E, Haupenthal J, Bönig H, et al Differential Stability of Cell-Free Circulating microRNAs: Implications for Their Utilization as Biomarkers. PLOS ONE. 2013;8(9):e75184 10.1371/journal.pone.0075184 24073250 PMC3779196

[65] Xiang M, Zeng Y, Yang R, Xu H, Chen Z, Zhong J, et al U6 is not a suitable endogenous control for the quantification of circulating microRNAs. Biochem Biophys Res Commun. 2014;454(1):210–4. Epub 2014/12/03. 10.1016/j.bbrc.2014.10.064 . 25450382

[66] Muth DC, Powell BH, Zhao Z, Witwer KW. miRNAs in platelet-poor blood plasma and purified RNA are highly stable: a confirmatory study. BMC research notes. 2018;11(1):273 Epub 2018/05/08. 10.1186/s13104-018-3378-6 29728133 PMC5936026

[67] Farina NH, Wood ME, Perrapato SD, Francklyn CS, Stein GS, Stein JL, et al Standardizing analysis of circulating microRNA: clinical and biological relevance. J Cell Biochem. 2014;115(5):805–11. 10.1002/jcb.24745 24357537 PMC3992702

