# Supplementary figures and images for: Impact of long-term storage and freeze-thawing on eight circulating microRNAs in plasma samples
Source: PLoS One. 2020 Jan 14;15(1):e0227648. doi: 10.1371/journal.pone.0227648 (PMC6959605; doi:10.1371/journal.pone.0227648)

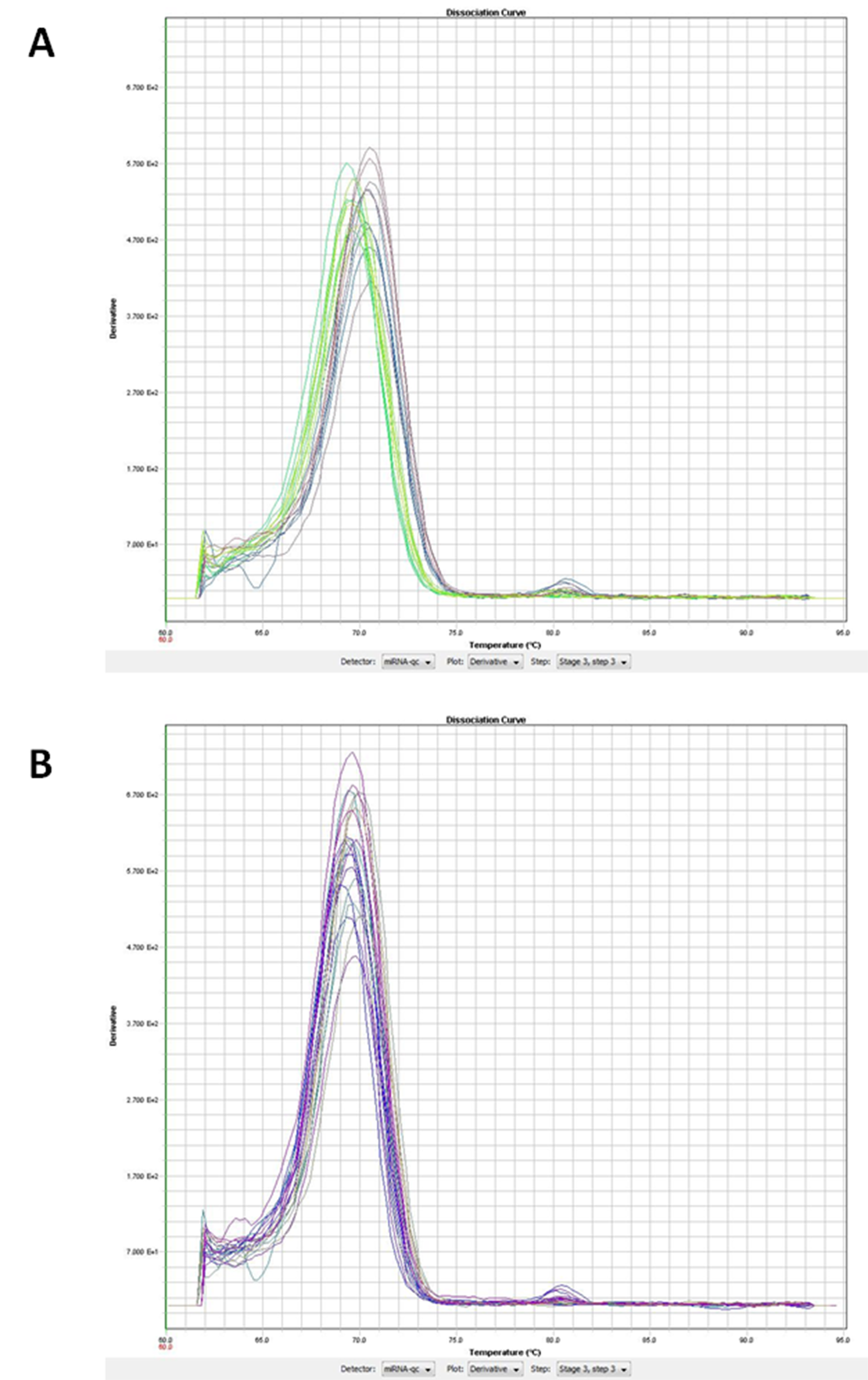

Supplement: S1 Fig — Panel A shows the melting curve analysis from the miR-30c-5p assay. Panel B shows the melting curve analysis from the miR-451a assay. (TIFF) [file pone.0227648.s001.tiff]

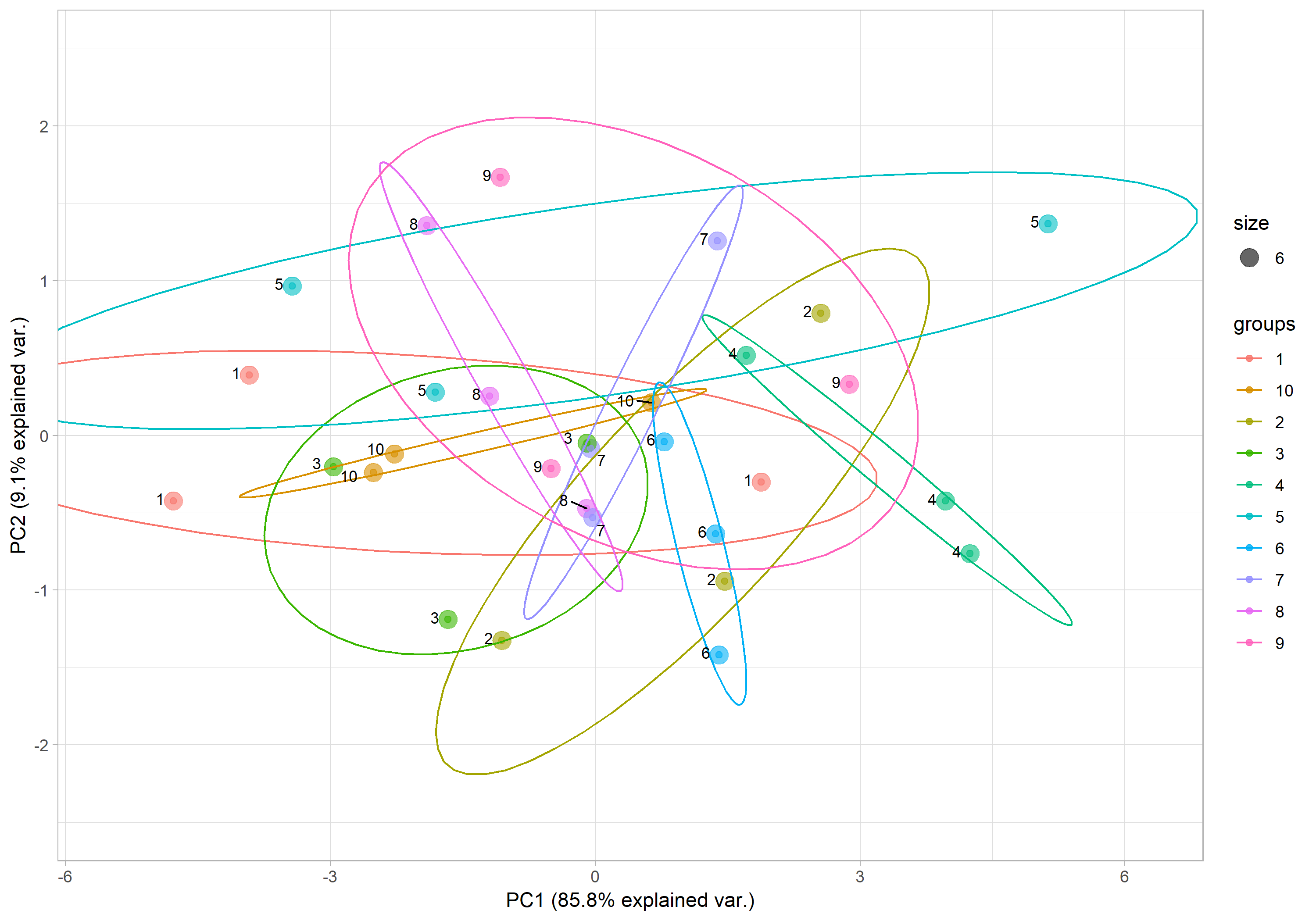

Supplement: S2 Fig — Scatterplot of the first two principal component scores from the PCA analysis of storage time data. Principal components one (shown on the x-axis) and two (y-axis) explain 85.80% and 9.10% of the data variance, respectively. Colors correspond to the ten subjects from the KORA study to facilitate visual inspection, and a confidence ellipse for the population mean was drawn around the grouped observations in order to visualize the variance (data with higher variance creates larger confidence ellipses). Observations from the three time points deriving from the same subject are labeled with an ordinal number corresponding to the subject. (TIFF) [file pone.0227648.s002.tiff]

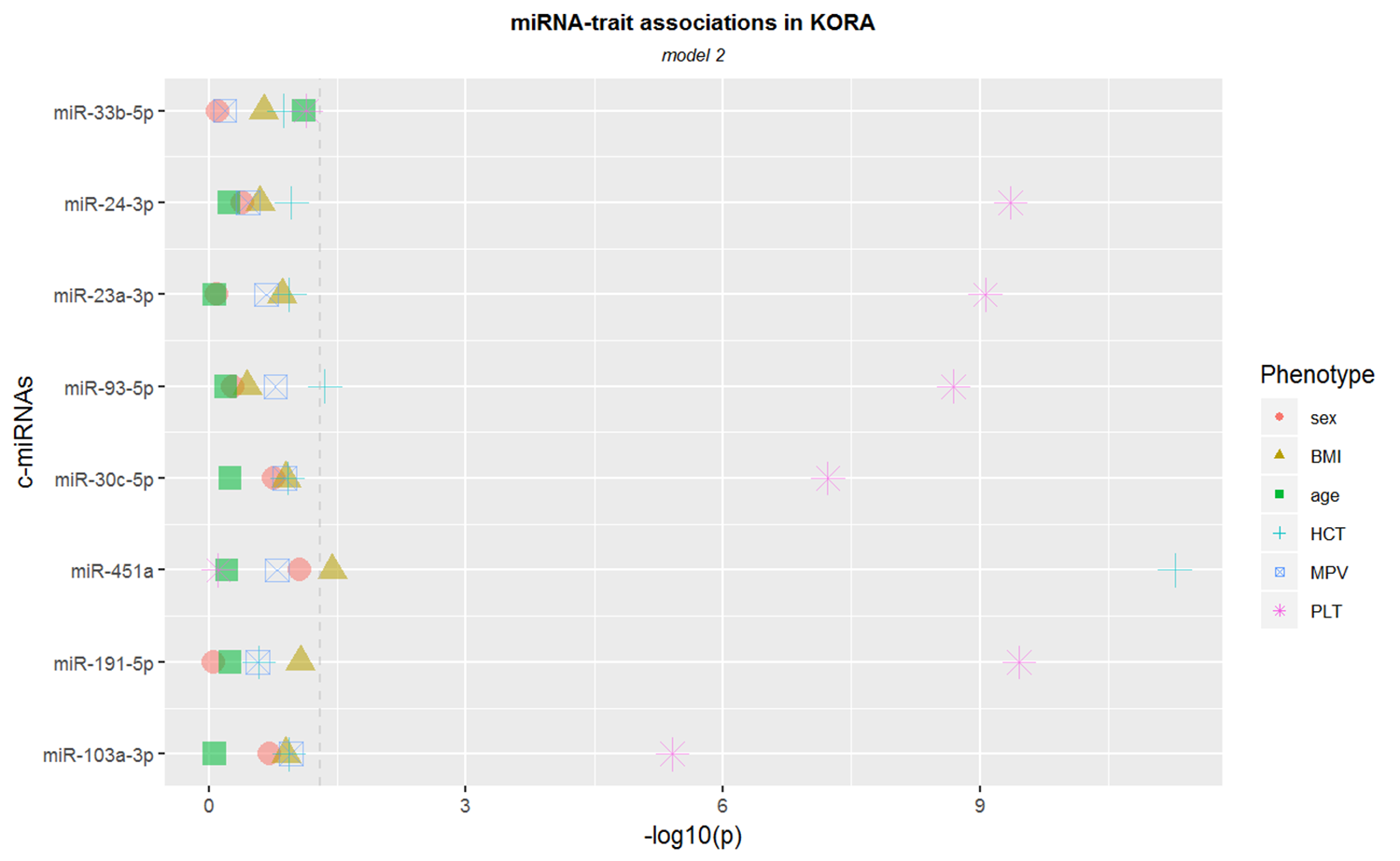

Supplement: S3 Fig — Dot plot with the p-value results from model 2 (miRNA level ~ age + sex + BMI + UniSp2 + ΔUniSp4—UniSp2 + Hct + platelets + MPV) as obtained from the linear regression analysis done with data from 300 participants from the KORA F4 survey. The negative logarithm of the p-value of the association between each miRNA and the covariates included in model 2 is plotted in the x-axis, while the y-axis lists the studied miRNAs. The different covariates are color and symbol coded. The results for the two technical parameters UniSp2 and Δ(UniSp4-UniSp2), corresponding to the oligonucleotides spiked-in during sample processing used as covariates in the model to adjust for technical variability [56], are not shown in this plot as to favor simplicity. (TIFF) [file pone.0227648.s003.tiff]

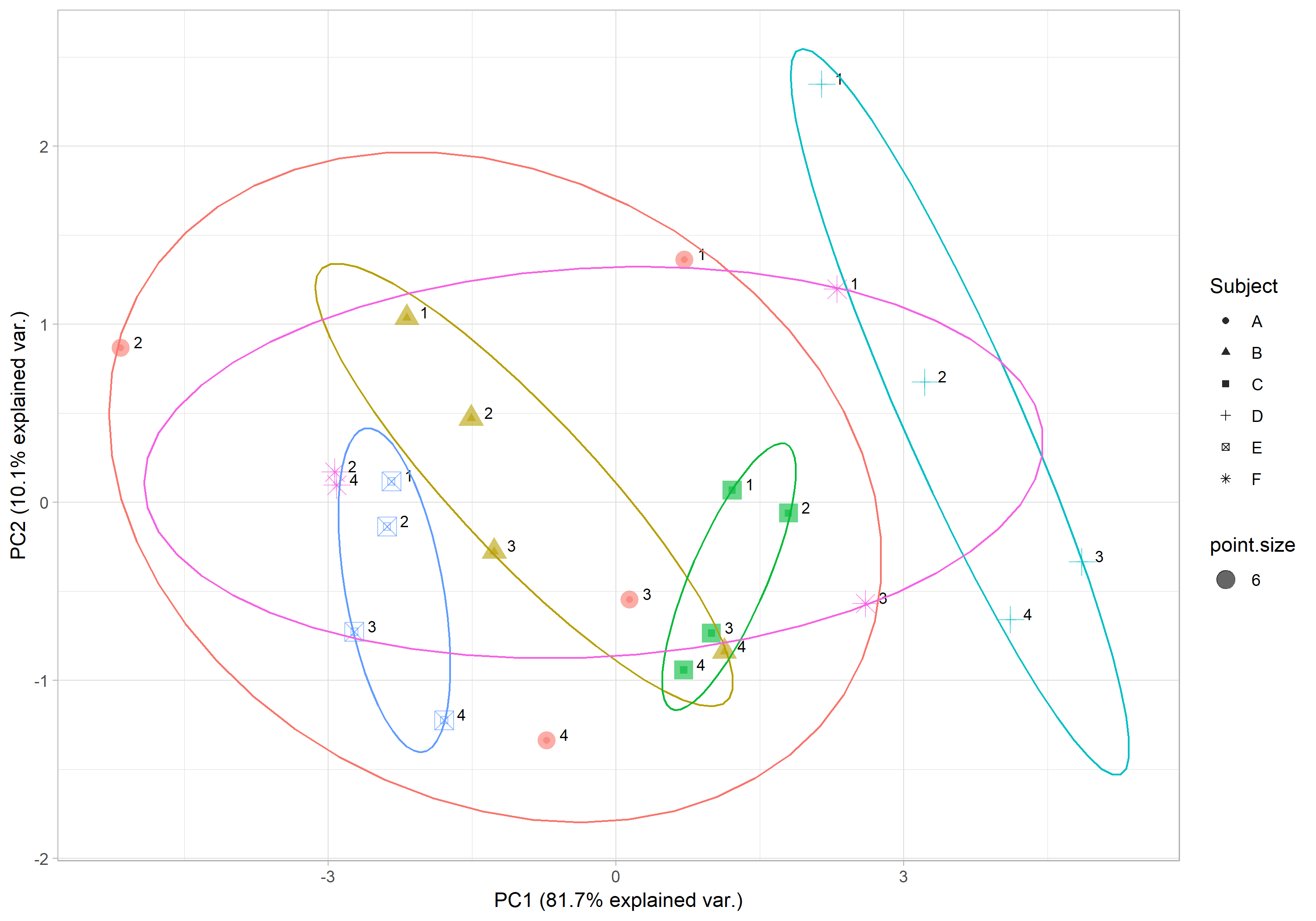

Supplement: S4 Fig — Scatterplot of the first two principal component scores from the PCA analysis of storage time data. Principal components one (shown on the x-axis) and two (y-axis) explain 81.70% and 10.10% of the data variance, respectively. Symbols and colors correspond to the six volunteers (subjects A to F) to facilitate visual inspection, and a confidence ellipse for the population mean was drawn around the grouped observations in order to visualize the variance (data with higher variance creates larger confidence ellipses). (TIFF) [file pone.0227648.s004.tiff]
