# Supplementary material for: Impact of long-term storage and freeze-thawing on eight circulating microRNAs in plasma samples
Source: PLoS One. 2020 Jan 14;15(1):e0227648. doi: 10.1371/journal.pone.0227648 (PMC6959605; doi:10.1371/journal.pone.0227648)
Supplement: S1 Table — The full nomenclature, accession numbers and sequences for the nine miRNAs studied in this project, as retrieved from miRbase, release 22. (DOC) [file pone.0227648.s005.doc]

| **Supporting Table S1. miRNA accession number. nomenclature and sequence.** | | | |
| --- | --- | --- | --- |
|  |  |  |  |
| **Accession number** | **Nomenclature** | **Sequence** |  |
| [MIMAT0000244](http://www.mirbase.org/cgi-bin/mature.pl?mature_acc=MIMAT0000244) | hsa-miR-30c-5p | UGUAAACAUCCUACACUCUCAGC |  |
| [MIMAT0000101](http://www.mirbase.org/cgi-bin/mature.pl?mature_acc=MIMAT0000101) | hsa-miR-103a-3p | AGCAGCAUUGUACAGGGCUAUGA |  |
| [MIMAT0000440](http://www.mirbase.org/cgi-bin/mature.pl?mature_acc=MIMAT0000440) | hsa-miR-191-5p | CAACGGAAUCCCAAAAGCAGCUG |  |
| [MIMAT0000422](http://www.mirbase.org/cgi-bin/mature.pl?mature_acc=MIMAT0000422) | hsa-miR-124-3p | UAAGGCACGCGGUGAAUGCCAA |  |
| [MIMAT0001631](http://www.mirbase.org/cgi-bin/mature.pl?mature_acc=MIMAT0001631) | hsa-miR-451a | AAACCGUUACCAUUACUGAGUU |  |
| [MIMAT0000078](http://www.mirbase.org/cgi-bin/mature.pl?mature_acc=MIMAT0000078) | hsa-miR-23a-3p | AUCACAUUGCCAGGGAUUUCC |  |
| [MIMAT0000093](http://www.mirbase.org/cgi-bin/mature.pl?mature_acc=MIMAT0000093) | hsa-miR-93-5p | CAAAGUGCUGUUCGUGCAGGUAG |  |
| [MIMAT0000080](http://www.mirbase.org/cgi-bin/mature.pl?mature_acc=MIMAT0000080) | hsa-miR-24-3p | UGGCUCAGUUCAGCAGGAACAG |  |
| [MIMAT0003301](http://www.mirbase.org/cgi-bin/mature.pl?mature_acc=MIMAT0003301) | hsa-miR-33b-5p | GUGCAUUGCUGUUGCAUUGC |  |
