# Supplementary material for: Impact of long-term storage and freeze-thawing on eight circulating microRNAs in plasma samples
Source: PLoS One. 2020 Jan 14;15(1):e0227648. doi: 10.1371/journal.pone.0227648 (PMC6959605; doi:10.1371/journal.pone.0227648)
Supplement: S2 Table — The table shows the results from the linear regression models used to analyze data from 300 participants from the KORA F4 study. miRNAs were profiled using the Exiqon Serum/Plasma Focus microRNA PCR Panel V3.M (Exiqon A/S) as described by the manufacturer’s protocol, and data went through the same quality control measures as described in the main Methods section. Information on BMI and the other covariates used in the models was collected at the time of interview within the KORA F4 survey. Two models were used: model 1, with age, sex, and BMI as predictor variables; and model 2, adding to those variables from model 1 technical variables (UniSp2 + ΔUniSp4—UniSp2) and blood parameters (hematocrit levels, Hct; platelet count; mean platelet volume, MPV) [56]. (DOCX) [file pone.0227648.s006.docx]

**Supporting Table S2. Cross-sectional analysis in KORA F4: results from linear regression models.**

**A**

|  | **Model 1** | | | | | | | | | |
| --- | --- | --- | --- | --- | --- | --- | --- | --- | --- | --- |
|  | **miRNA level ~ age + sex + BMI** | | | | | | | | | |
| **Covariates** | **Age** | | | **Sex** | | | **BMI** | | | **N** |
| **miRNA** | **Estimate** | **SE** | **P value** | **Estimate** | **SE** | **P value** | **Estimate** | **SE** | **P value** |  |
| miR-103a-3p | -0.016 | 0.017 | 0.353 | -0.462 | 0.157 | 0.003 | -0.013 | 0.016 | 0.427 | 285 |
| miR-191-5p | -0.004 | 0.014 | 0.787 | -0.283 | 0.124 | 0.024 | -0.009 | 0.013 | 0.501 | 285 |
| miR-451a | -0.017 | 0.014 | 0.218 | -0.093 | 0.128 | 0.469 | 0.008 | 0.013 | 0.559 | 285 |
| miR-30c-5p | -0.008 | 0.015 | 0.586 | -0.434 | 0.135 | 0.001 | -0.009 | 0.014 | 0.533 | 285 |
| miR-93-5p | -0.009 | 0.013 | 0.488 | -0.309 | 0.118 | 0.009 | -0.002 | 0.012 | 0.843 | 285 |
| miR-23a-3p | -0.010 | 0.013 | 0.409 | -0.279 | 0.116 | 0.017 | -0.006 | 0.012 | 0.586 | 285 |
| miR-24-3p | -0.006 | 0.013 | 0.651 | -0.334 | 0.119 | 0.005 | -0.003 | 0.012 | 0.785 | 282 |
| miR-33b-5p | 0.004 | 0.022 | 0.873 | -0.476 | 0.203 | 0.019 | -0.018 | 0.021 | 0.371 | 285 |

**B**

|  | **Model 2** | | | | | | | | | |
| --- | --- | --- | --- | --- | --- | --- | --- | --- | --- | --- |
|  | **miRNA level ~ age + sex + BMI + UniSp2 + Δ UniSp4 - UniSp2 + Hct + platelets + MPV** | | | | | | | | | |
| **Covariates** | **Age** | | | **Sex** | | | **BMI (kg/m^2^)** | | | **N** |
| **miRNA** | **Estimate** | **SE** | **P value** | **Estimate** | **SE** | **P value** | **Estimate** | **SE** | **P value** |  |
| miR-103a-3p | -0.002 | 0.014 | 0.867 | -0.185 | 0.143 | 0.196 | -0.020 | 0.013 | 0.125 | 284 |
| miR-191-5p | 0.006 | 0.011 | 0.563 | -0.014 | 0.109 | 0.896 | -0.017 | 0.010 | 0.086 | 284 |
| miR-451a | -0.005 | 0.010 | 0.613 | -0.172 | 0.100 | 0.087 | 0.019 | 0.009 | 0.036 | 284 |
| miR-30c-5p | 0.006 | 0.010 | 0.562 | -0.145 | 0.107 | 0.176 | -0.015 | 0.010 | 0.125 | 284 |
| miR-93-5p | 0.004 | 0.009 | 0.636 | -0.059 | 0.092 | 0.521 | -0.008 | 0.008 | 0.358 | 284 |
| miR-23a-3p | 0.002 | 0.009 | 0.865 | -0.024 | 0.094 | 0.801 | -0.013 | 0.009 | 0.140 | 284 |
| miR-24-3p | 0.005 | 0.010 | 0.579 | -0.083 | 0.099 | 0.402 | -0.010 | 0.009 | 0.254 | 281 |
| miR-33b-5p | 0.030 | 0.017 | 0.077 | 0.049 | 0.175 | 0.779 | -0.019 | 0.016 | 0.230 | 284 |
